# Hyperoxia does not improve the acute upper thermal tolerance of a tropical marine fish (*Lutjanus apodus*)

**DOI:** 10.1242/jeb.247703

**Published:** 2024-11-07

**Authors:** Rebeccah M. Sandrelli, Emma S. Porter, Anthony K. Gamperl

**Affiliations:** Department of Ocean Sciences, Memorial University of Newfoundland and Labrador, St. John's, NL, Canada A1C 5S7

**Keywords:** Critical thermal maximum, Heart function, Metabolism, Climate change, Tropical fish, Aerobic scope, Water oxygen level

## Abstract

Fish can experience hyperoxia in shallow environments due to photosynthetic activity and this has been suggested to provide them with a metabolic refuge during acute warming. However, this hypothesis has never been tested on a tropical marine species. Thus, we fitted 29°C-acclimated wild schoolmaster snapper (*Lutjanus apodus*; a species known to experience diel hyperoxia in mangrove creeks and coastal waters) with Transonic^®^ flow probes and exposed them to an acute increase in temperature (at 1°C h^−1^) in respirometers under normoxia and hyperoxia (150% air saturation), until their critical thermal maximum (CT_max_). The CT_max_ of both groups was ∼39°C, and no differences in maximum cardiac function were recorded as the fish were warmed. However, temperature-induced factorial aerobic scope was significantly greater in fish tested under hyperoxia. These data suggest that hyperoxia will not protect coastal tropical fish species during marine heat waves, despite its effects on metabolic scope/capacity.

## Teaser

**Summary:** Hyperoxia improves the metabolic scope of schoolmaster snapper when acutely warmed, but not their upper thermal tolerance.

## INTRODUCTION

Climate change is a major threat to fishes given that: (1) temperature has a major influence on the biology and physiology of aquatic ectotherms (Brett, 1971); (2) average ocean temperatures are predicted to rise 2–4°C by 2100 (IPCC, 2022); and (3) warming events (i.e. heat waves) are becoming more frequent and severe (Collins et al., 2019; Frölicher et al., 2018; Holbrook et al., 2019; IPCC, 2022; Oliver et al., 2018). Thus, it is not surprising that ecophysiologists are examining the impacts of temperature changes on the biology of important fishes. However, such studies have been largely focused on temperate and polar (mainly Antarctic) species, and little has been done to understand how tropical/subtropical species will be affected by climate change even though there is clear evidence of this phenomenon's impact (e.g. see Genin et al., 2020).

**List of abbreviations**

AS_T_: temperature-induced absolute aerobic scope
CT_max_: critical thermal maximum
FAS_T_: temperature-induced factorial aerobic scope
*f*_H_: heart rate
LT_50_: temperature that is lethal to 50% of a population
MMR_T_: temperature-induced maximum metabolic rate
*Ṁ*_O_2__: oxygen uptake
*Ṁ*_O_2__/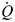
tissue oxygen extraction
*P*v_O_2__: partial pressure of oxygen in the venous blood
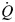
cardiac output
RMR: resting metabolic rate
RVM: relative ventricular mass
SMR: standard metabolic rate
*V*_s_: stroke volume

The Tropical Marine Ecophysiology Laboratory (TMEP-Lab) was recently established on Cape Eleuthera (The Bahamas), with the goals of performing long-term measurements of water conditions in various marine habitats, and conducting experiments on various fish and invertebrate species to understand how climate change-related alterations in environmental parameters may/will affect their physiology. This type of research is critical if we are to understand how acute and chronic changes in environmental parameters will influence the physiology of ecologically and economically important fish species, and management and conservation efforts are to be effective in the era of climate change (e.g. see Little et al., 2020; Seebacher et al., 2023).

**Fig. 1.**
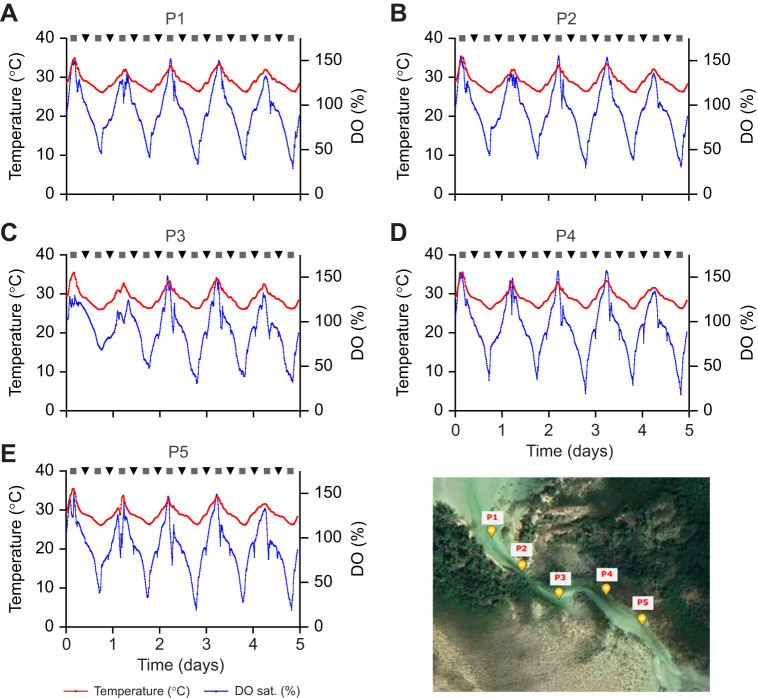
**Temperature (°C) and oxygen (% air saturation) data collected in Page Creek (Eleuthera, The Bahamas) in October 2022.** Data were collected every 10 min for 5 days using miniDOT Clear^Ⓡ^ loggers (Precision Measurement Engineering, Inc., Vista, CA, USA) placed every 50 m (P1–P5) from the mouth of the creek. The panel in the bottom right corner shows the placement of the loggers within the creek. ▪ and ▾ indicate high and low tides, respectively. Both temperature and water oxygen levels peaked at ∼14:00–15:00 h.

One of the ecosystems under study by the TMEP-Lab are mangrove creeks, as these shallow habits (like others with considerable algal populations; McArley et al., 2021a) are characterized by diel temperature and oxygen cycles, with high temperatures and hyperoxia occurring during daylight hours (Fig. 1). Mangroves are a key foraging habitat and nursery ground for many tropical marine species; however, it is unclear from the research conducted to date whether hyperoxia (up to 150% air saturation) in Bahamian mangrove creeks might provide these fishes with a metabolic refuge during acute warming. Hyperoxic conditions can increase fish aerobic capacity and heart performance at high temperatures (Brijs et al., 2015; Ekström et al., 2016; McArley et al., 2018, 2021a, 2022a) and in some cases, upper thermal tolerance limits (Ekström et al., 2016; Giomi et al., 2019; McArley et al., 2018, 2021a, 2022a). However, the above studies have almost exclusively been done on temperate fish species, and while McArley et al. (2018) demonstrated that hyperoxia significantly increased the thermal tolerance of the less thermally tolerant triplefin (*Forsterygion lapillum*), no such effect was seen for the more thermally tolerant *Bellapiscis medius*. Moreover, while European perch (*Perca fluvialitis*) from typical Baltic Sea areas (‘reference’ fish) have an increased upper thermal tolerance when hyperoxic (Ekström et al., 2016), populations from the Biotest enclosure (a unique coastal ecosystem that maintains natural thermal fluctuations but has been warmed by 5–10°C by warm water discharge from a nuclear power plant for over three decades) showed no hyperoxia-related improvement in upper thermal limits (Brijs et al., 2015). Collectively, these latter data suggest that hyperoxia will not provide tropical fish species with a metabolic refuge during acute warming events.

Hyperoxia occurs in many shallow aquatic habitats, and thus, as pointed out by McArley et al. (2021a), further research establishing the ecophysiological implications of concomitant heat stress and hyperoxia is needed, particularly with respect to naturally occurring hyperoxic episodes and in light of climate change. Thus, we exposed schoolmaster snapper (*Lutjanus apodus*) to a critical thermal maximum (CT_max_) challenge at an ecologically relevant heating rate (1°C h^−1^; see Fig. 1) when exposed to normoxia and 150% air saturation, while measuring their oxygen consumption (*Ṁ*_O_2__) and cardiac function. This is because aerobic scope (the amount of oxygen consumption/aerobic-based metabolism available above that required to support essential physiological functions; Fry, 1971; Pörtner et al., 2017; Pörtner and Knust, 2007; Schulte, 2015) and cardiac function [heart rate (*f*_H_), cardiac output (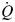) and stroke volume (*V*_s_)] (Eliason et al., 2013; Ern et al., 2023; Farrell, 2009; Vornanen, 2020; Wang and Overgaard, 2007) are thought to be central to understanding a fish's performance at a given temperature; although, to what extent aerobic scope (AS) is related to a fish's thermal tolerance is being actively debated (Clark et al., 2013; Jutfelt et al., 2018; Ern et al., 2016, 2023). The schoolmaster snapper was chosen for these studies as: (1) it is a widely distributed marine species (i.e. it is native to the Caribbean, the Gulf of Mexico and the northeastern coast of South America); (2) this species inhabits mangroves and shallow patch reefs until they mature; and (3) mangroves in the Bahamas can experience temperatures as high as 40°C coincident with hyperoxia in the summer (Porter and Gamperl, 2023; Schneider et al., 2023; Fig. 1). Thus, it is an ideal species to test the hypothesis that fish naturally exposed to hyperoxia at high environmental temperatures (i.e. tropical species), will not show any improvements in temperature-induced metabolic and cardiac function or thermal tolerance when tested under hyperoxic versus normoxic conditions.

## MATERIALS AND METHODS

All experimental work described was approved by the Institutional Animal Care Committee of Memorial University of Newfoundland (protocol 22-01-KG) and the Government of The Bahamas' Departments of Environmental Planning and Protection and Marine Resources (Research Permit BS-2022-873637), and followed the standards and guidelines outlined by the Canadian Council on Animal Care (www.ccac.ca).

### Fish husbandry

Schoolmaster snapper (*Lutjanus apodus* Walbaum 1792) 239.8±15 g (range 137–327 g) and 25.0±0.5 cm fork length (range 22.0–28.5 cm) were collected using baited traps within 20 m of shore in the vicinity of the Cape Eleuthera Institute (CEI) in Eleuthera (The Bahamas) in October of 2022. They were then transported to the wet lab facility at CEI and held in round tanks (1.6 m^3^) supplied with seawater pumped from ∼30 m offshore (temperature ∼28.5–30°C). The fish were fed cut up sardines (*Sardinella aurita*) and conch (*Aliger gigas*) at a ration of ∼2.5% body weight on alternate days and exposed to an ambient photoperiod.

### Surgery and overnight oxygen consumption measurements

Each fish was netted from their holding tank and anaesthetized in buffered (NaHCO_3_) seawater containing tricaine methansulfonate (Syncaine TMS, 0.2 g l^−1^) until ventilatory movements ceased. The fish were weighed and measured for fork length, and placed on their right side on a wetted foam pad on a surgical table while their gills were irrigated continuously with ∼29°C oxygenated seawater containing a maintenance dose of TMS (0.1 g l^−1^). Then, umbilical tape was passed under the gill arches and secured to the surgical table to allow access to the opercular cavity. A small puncture was then made just below the base of the 4th gill arch with a pair of Dumont forceps, and the ventral aorta was carefully located using blunt dissection. Once identified, the ventral aorta was freed from the surrounding tissue using a pair of curved forceps without damaging the pericardium, and a Transonic^®^ flow probe (Model RB Transducer, 1.5 mm in diameter; Transonic Systems, Ithaca, NY, USA) was fitted around the ventral aorta. Finally, the flow probe lead was connected to a transit time perivascular flowmeter (Model TS420/T20; Transonic Systems Inc.) to ensure that the signal was of high quality and secured to the fish at three locations using 3-0 silk suture; one suture immediately ventral to the pectoral fin, one just below the lateral line and one in front of the dorsal fin.

After surgery was completed (15–20 min.), individual fish were placed in a ∼7.0 l cylindrical respirometer (14 cm in diameter×45 cm long) submerged in a shallow water table containing fully aerated seawater at 29°C. The respirometers holding the fish (*n*=2 per trial) were each equipped with a flush pump and a recirculation pump (both Eheim 5 l min^−1^, Model 1046; Eheim Gmbh & Co., Deizisau, Germany). The recirculation pump was placed in a loop consisting of Nalgene gas-tight tubing (ThermoScientific, Waltham, MA, USA) and recirculated water through the chamber (see below). The fish were allowed to recover/acclimate inside the respirometers for ∼20 h (i.e. until the next morning) at 29°C. Overnight, resting *Ṁ*_O_2__ measurements were made (see below) every 15 minutes from 18:00–06:00 h. During this period, oxygen consumption (*Ṁ*_O_2__; in mg O_2_ kg^−1^ h^−1^) was measured using intermittent closed respirometry with 11 min flush, 2 min wait and 3 min measurement periods (Killen et al., 2021; Rodgers et al., 2016; Svendsen et al., 2016). The flush pump and recirculation pump were controlled by SmartShifter software (Norin and Gamperl, 2018) and were intermittently turned on or off to either flush the respirometer with fresh seawater or create a sealed respirometry chamber when off. *Ṁ*_O_2__ recordings were made using a Firesting fiber-optic oxygen meter fitted with calibrated dipping probes (Pyroscience, Archen, Germany) that were inserted inside the respirometers, and the output from this meter was recorded on a computer running Pyro Oxygen Logger software. The rate of oxygen decline during the closed phase of the respirometry cycle (i.e. when the flush pump was off) was used to calculate the *Ṁ*_O_2__ of the fish.

### Experimental protocol

The lab's lights were turned on at 06:00 h (although it was already daylight) and the flow probe leads were connected to the flow meter; the signal from the flow meter amplified and filtered using a data acquisition system (MP160; BIOPAC Systems, Inc., Santa Barbara, CA, USA) and a universal interface module (UIM100A, BIOPAC Systems, Inc.; Goleta, CA, USA) and recorded by the same computer running AcqKnowledge^®^ software (Version 5.0; BIOPAC Systems, Inc.). One hour later, oxygen was either: (1) maintained at 100% air saturation or (2) increased to 150% air saturation over 1 h (*N*=9 per group). Water oxygen levels were maintained or increased by bubbling pure oxygen (gas) into the water table. The addition of oxygen was controlled by a second computer running WitroxCTRL^®^ software (Loligo Systems, Viborg, Denmark) that was interfaced with a fibre optic O_2_ meter (Witrox-1) with O_2_ dipping probe (Loligo Systems, Viborg, Denmark) and solenoid valves connected to an oxygen cylinder.

An acute upper thermal challenge at 1°C h^−1^ to the fish's critical thermal maximum (CT_max_) was used to determine the schoolmaster snapper's upper thermal tolerance; i.e., the temperature at which the fish lost equilibrium. This temperature increase was achieved using a 1000 W and an 1800 W submersible heater (Intelligent Heater LLC, GA, USA), and manually changing the set points. Cardiac function and *Ṁ*_O_2__ measurements were taken at each 1°C increment, with *Ṁ*_O_2__ calculated over the final 5 min of the 7 min closed period. Thereafter, the fish were euthanized with 0.4 g l^−1^ TMS and ventricular mass (g) was recorded.

### Data and statistical analyses

Standard metabolic rate (SMR) was calculated as both the lowest 10 and 20% of the *Ṁ*_O_2__ measurements from the overnight period. Resting (routine) metabolic rate (RMR) and MMR were recorded as the *Ṁ*_O_2__ just prior to temperature being increased and the highest (maximum) metabolic rate measured for each fish when warmed to their CT_max_ (MMR_T_), respectively. Temperature-induced aerobic scope (AS_T_) was calculated as the difference between MMR_T_ and RMR, while temperature-induced factorial aerobic scope (FAS_T_) was calculated at MMR_T_/RMR. Background measurements of *Ṁ*_O_2__ were made after each fish was tested, and these were negligible (<1%), indicating that no substantial microbial respiration was occurring (Rodgers et al., 2016; Svendsen et al., 2016). It should be noted that the maximum metabolic rate (MMR) measured during the CT_Max_ test likely underestimate that achieved during other metabolic demanding challenges such as a critical swimming speed (*U*_crit_) test (Eisenberg et al., 2024; Nati et al., 2024; Norin et al., 2019). However, the values of MMR and AS measured during this test represent the metabolic capacity that a fish has available when faced with an acute increase in temperature. To be clear about what we measured, we refer to ‘temperature-induced’ MMR, AS and FAS (MMR_T_, AS_T_ and FAS_T_).

Heart rate (*f*_H_; in beats min^−1^) was measured by determining the average time required for 20 systolic peaks in the blood flow trace (5 segments analysed per fish) while the system was closed for respirometry, and values for cardiac output (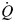; the amount of blood pumped by the heart) were recorded in ml min^−1^ kg^−1^. This allowed for stroke volume (*V*_S_; the amount of blood pumped per heartbeat) to be calculated as 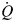/*f*_H_ (in units of ml kg^−1^) and tissue oxygen extraction to be calculated as *Ṁ*_O_2__/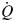 (in mg O_2_ ml blood^−1^). Note that the Transonic^®^ flow probes were calibrated using saline and ∼10% haematocrit over a range of temperatures (20–40°C) to ensure that flow values measured in fish at the two temperatures were accurate. Relative ventricular mass (RVM) was calculated as (ventricular mass/fish mass)×100.

A Rosner's Test [*EnSVtats* package in R (CRAN.R-project.org/package=EnvStats) with α=0.05 (Millard, 2013)] and a Grubb's Test [*outliers* package in R with α=0.05 (CRAN.R-project.org/package=outliers; komsta.net)] were used to examine if there were outliers in all datasets prior to statistical analysis. However, none were identified. All data were then tested for assumptions of normality and homogeneity of variance using Shapiro–Wilks and Levene's tests, respectively. A Welch's two sample *t*-test was used (*stats* package in R) to compare all morphometric, cardiac and metabolic data between the groups. All statistical analyses were performed using Rstudio v.2022.12.0+353 with R v.4.2.3 (r-project.org), and all data in the text, figures and tables are means±s.e.m. The threshold used for determining statistical significance was *P*<0.05.

## RESULTS AND DISCUSSION

To our knowledge, only one study has previously investigated the effects of hyperoxia on both oxygen consumption/aerobic capacity and cardiac function (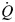, *V*_S_ and *f*_H_) as affected by acute warming in fishes (McArley et al., 2022a). Furthermore, the present study is the first to examine the influence of elevated oxygen levels on the cardiorespiratory physiology of a subtropical/tropical marine fish species. Thus, the data presented here have important implications for how these combined environmental conditions influence fish physiology, but also with regard to the potential impacts of climate change-related heat waves on the survival of fishes that inhabit mangroves and shallow patch reefs.

Most resting (initial) cardiorespiratory parameters were similar between the normoxic and hyperoxic groups. For example, RMR was ∼210 mg O_2_ kg^−1^ h^−1^ and resting 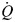, *V*_S_ and *Ṁ*_O_2__/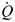 were ∼32–40 ml kg^−1^ min^−1^, 0.37 ml beat^−1^ and ∼0.1 mg O_2_ ml blood^−1^, respectively. However, resting *f*_H_ was approximately 17 beats min^−1^ (∼16.5%) less in the hyperoxic group and this difference between groups was very close to being significant (*P*=0.07) (Table 1). That SMR and RMR were not influenced by hyperoxia in this experiment is consistent with the majority of the literature. RMR has been shown to either not be affected by hyperoxia or to only increase slightly (by ∼10%) (McArley et al., 2018, 2021b and 2022a,b). However, Skeeles et al. (2022) recently reported a decrease in SMR (by ∼18%) in the common galaxias (*Galaxias maculatus*) and suggested that this may have been related to a decreased cost of ventilation. A lower *f*_H_ in hyperoxic fish would be consistent with the findings of Holeton (1972) for blackfin icefish (*Chaenocephaltis aceratus*) and is likely to be mediated by an increased cholinergic tone on the heart (Wilkes et al., 1981). However, there are several other studies that have reported no effect of hyperoxia on resting *f*_H_ or other cardiac parameters (Berschick et al., 1987; Reid et al., 2005; Ekström et al., 2016; McArley et al., 2021b, 2022b). Nonetheless, it is difficult to interpret our data as there are only 13 studies that have examined the effect of hyperoxia on *f*_H_, and even fewer (three) have measured the effects of elevated water O_2_ levels on 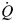.

**Table 1.**
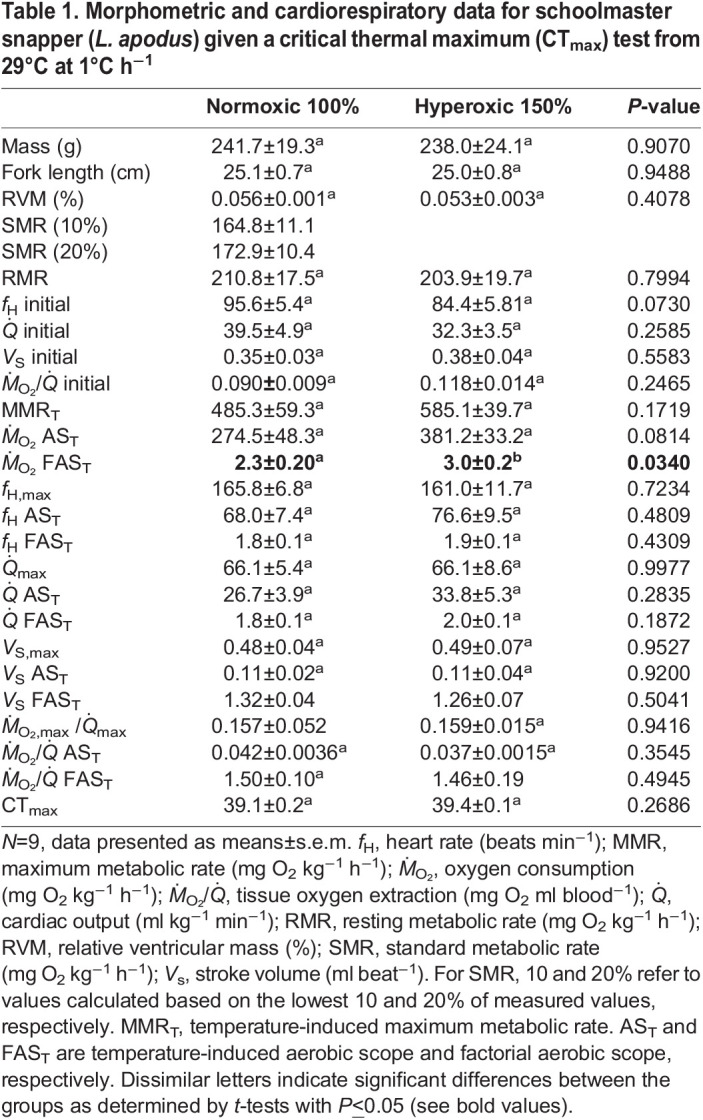
Morphometric and cardiorespiratory data for schoolmaster snapper (*L. apodus*) given a critical thermal maximum (CT_max_) test from 29°C at 1°C h^−1^

**Fig. 2.**
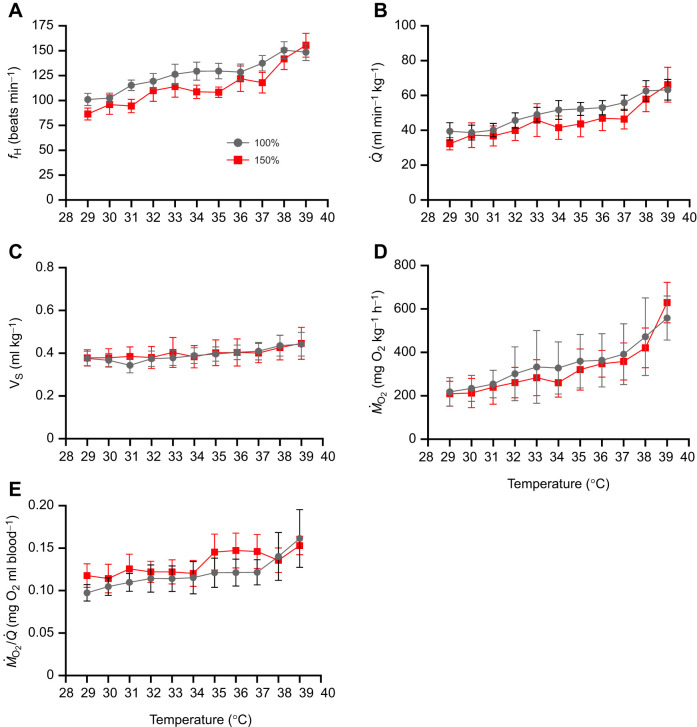
**Cardiorespiratory function in schoolmaster snapper (*Lutjanus apodus*) exposed to an incremental increase in temperature from their acclimation temperature (29°C) at 1°C h^−1^ until the fish reached their critical thermal maximum (CT_max_).** These CT_max_ tests were conducted both under normoxic (100% air saturation) and hyperoxic (150% air saturation) conditions. Air saturation of 150% was chosen as this was the highest oxygen level measured in Page Creek, Cape Eleuthera, The Bahamas (see Fig. 1). *N*=9, data presented as means±s.e.m. *f*_H_, heart rate; 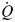, cardiac output; *V*_S_, stroke volume; *Ṁ*_O_2__, oxygen consumption; *Ṁ*_O_2__/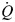, tissue oxygen extraction. Note: the data, plotted as box plots, are shown in Fig. S1 for transparency.

In both experimental groups, *Ṁ*_O_2__ appeared to increase in an exponential fashion until the snapper reached their CT_max_ (Fig. 2D). This higher *Ṁ*_O_2__ was largely the result of a ∼2-fold increase in 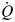 that was due solely to tachycardia; *V*_S_ did not change with temperature (Fig. 2A–C, Table 1). However, blood oxygen extraction (*Ṁ*_O_2__/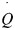, Fig. 2E) also increased by 50% as the normoxic fish were warmed. These temperature-induced responses in *Ṁ*_O_2__, 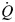, *f*_H_ and *V*_S_ are consistent with what has been observed in other teleost fish species (Farrell, 2009). An increase in *Ṁ*_O_2__/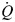 has also been reported in several other studies (Claësson et al., 2016; Clark et al., 2012; Leeuwis et al., 2021; Motyka et al., 2017) and our data provide further evidence that this parameter can play an important role in meeting the metabolic demands of fishes at high temperatures. In addition, our data support the hypothesis put forward by Leeuwis et al. (2021) that ṀO_2_ /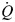 is predominantly used to meet increased metabolic demands in fish that have a limited maximum *f*_H_ and scope for *f*_H_. In contrast to the sablefish (*Anoplopoma fimbria*) and European eel (*Anguilla anguilla*), which have *f*_H,max_ values of <80 beats min^−1^ and an *f*_H_ scope <50 beats min^−1^ (and temperature-dependent increases in *Ṁ*_O_2__/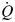 are∼2-fold) (Claësson et al., 2016; Leeuwis et al., 2021), the schoolmaster snapper has an *f*_H,max_ of 160 beats min^−1^ and a scope for *f*_H_ of 60–80 beats min^−1^. These latter parameters are very similar to those measured in salmonids like the Atlantic salmon (*Salmo salar*) and rainbow trout (*Oncorhynchus mykiss*), and these species also show limited increases in *Ṁ*_O_2__/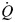 when actively exposed to increases in temperature (Clark et al., 2012; Moytka et al., 2017; Penney et al., 2014; Steinhausen et al., 2008). Clearly, however, this hypothesis needs to be tested on a number of species in one study and where the arterio-venous O_2_ difference is also directly measured.

The pattern of change in cardiorespiratory parameters was similar in the two groups, and there were no differences in the maximum values or scope for cardiac parameters (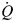, *f*_H_ and *V*_S_) or *Ṁ*_O_2__/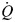 in fish tested under hyperoxia versus normoxia (Table 1 and Fig. 2). That maximum 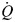 and *V*_S_ were not affected by hyperoxia is in contrast to Ekström et al. (2016) and McArley et al. (2022a) who reported that both these parameters were higher (by ∼30–50%) in hyperoxic European perch (*Perca fluviatalis*) and rainbow trout, respectively, prior to reaching their CT_max_. Furthermore, this result is surprising as FAS_T_ was significantly higher (3.0±0.2 vs 2.3±0.2; *P*=0.03) in snapper tested under hyperoxia versus normoxia (for AS_T_, *P*=0.08; Table 1) and we also found no increase in *Ṁ*_O_2__/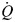 at higher temperatures or in the scope values for this parameter. That *Ṁ*_O_2__/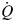 did not increase in fish given a CT_max_ test when hyperoxic is consistent with the data of McArley et al. (2022a), and with the fact that haemoglobin is normally fully saturated with oxygen under normoxic conditions (Perry and Reid, 1992). In addition, it is possible that the lack of an increase in 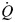 and *V*_S_ in warmed hyperoxic versus normoxic fish was due to *P*v_O_2__ not being different between the groups. While this hypothesis would not be consistent with the data presented in McArley et al. (2022a) and Ekström et al. (2016) for ‘reference’ perch, perch from the Biotest area had values for *P*v_O_2__ that were intermediate between those of normoxic and hyperoxic ‘reference’ perch as they were warmed, and equivalent to those of hyperoxic ‘reference’ fish at high temperatures. Thus, it is possible that thermal niche/history and the fact that schoolmaster snapper live in areas where both high temperature and hyperoxia are common temporal features, limits their effects on *V*_S_ and 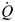 (i.e. they have reduced physiological plasticity with regard to these environmental conditions).

Despite the increases in FAS_T_ (30%; *P*=0.03) and AS_T_ (by 40%; *P*=0.08) in hyperoxic versus normoxic schoolmaster snapper, the difference in CT_max_ between the two groups of fish was only ∼0.3°C and not significantly different (*P*=0.27; Table 1). This result is consistent with approximately 50% of previous studies that have examined the influence of hyperoxia on the CT_max_ of fishes (McArley et al., 2021a, 2022a) and not surprising given that in studies that do report significant increases in CT_max_ the mean difference in this parameter is only 0.78°C (McArley et al., 2022a). Collectively, these studies; (1) infer that while hyperoxia may provide tropical fish species with a ‘metabolic refuge’ at sublethal warm temperatures and allow them to sustain aerobically demanding processes such as locomotion, growth and digestion (McArley et al., 2021a), it does not translate into increased thermal tolerance; and (2) provide additional data to refute the relevance of the oxygen and capacity limited thermal tolerance (OCLTT) hypothesis (Pörtner and Knust, 2007; Pörtner et al., 2017) with regard to short-term (acute) increases in temperature.

This former conclusion is in contrast to that of Giomi et al. (2019) who reported that two species of tropical fish (*Athernomorus* sp. and *Dasyllus* sp.) had temperature values at 50% mortality (LT_50_ values) that were 1.4 and 1.8°C higher, respectively, in hyperoxic (140% air saturation) fish. There may be two reasons for this discrepancy. First, the implications of the data in Giomi et al. (2019) are open to interpretation. The reported LT_50_ values were based on exposing each group of fish (normoxic versus hyperoxic) to a single temperature increase (i.e. *n*=1), and thus, no statistical analysis of this data was possible. Second, it is possible that surgery and confinement of the snappers in respirometers, influenced the CT_max_ values obtained in this study and the effects on hyperoxia on this parameter. Sandrelli and Gamperl (2023) recently highlighted the limitations of lab-based methods of determining *f*_H_ parameters and the thermal tolerance of fishes. Clearly, more research is needed on tropical fishes that inhabit coastal environments, and which reach high temperatures during the daytime, to determine whether hyperoxic environments (seagrass meadows, coral reefs, algal stands, and/or highly productive microbial mats associated with mangroves) offer a true thermal refuge for these fishes. Such information is critical given the ecological importance of these species, that fish such as the schoolmaster snapper are already experiencing temperatures close to their thermal limits (Nati et al., 2024; Schneider et al., 2023) and the predicted increases in average ocean temperature and in the severity and frequency of heat waves (IPCC, 2022).

## Supplementary Material

10.1242/jexbio.247703_sup1 Supplementary information
