# Supplementary material for: Hyperoxia does not improve the acute upper thermal tolerance of a tropical marine fish (Lutjanus apodus)
Source: J Exp Biol. 2024 Nov 7;227(21):jeb247703. doi: 10.1242/jeb.247703 (PMC11574356; doi:10.1242/jeb.247703)
Supplement: Supplementary information [file jexbio-227-247703-s1.pdf]

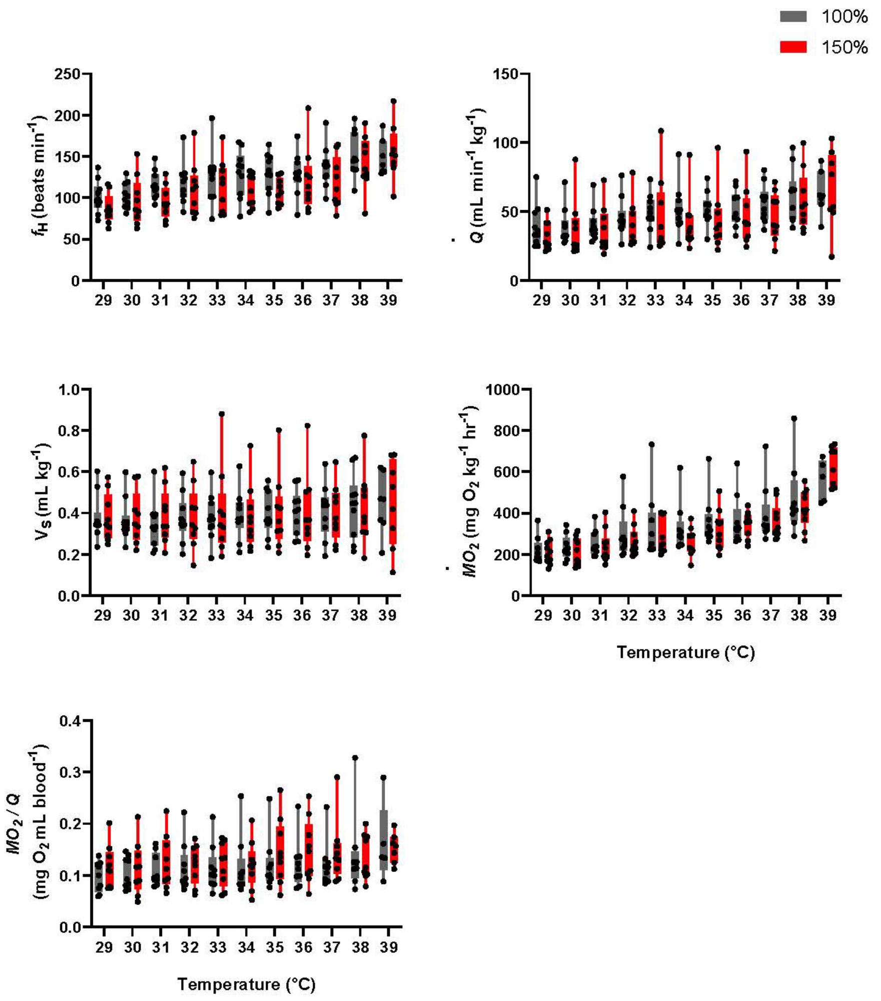

Fig. S1. Cardiorespiratory function is schoolmaster snapper (*L. apodus*) exposed to an incremental increase in temperature from their acclimation temperature (29°C) at 1°C h^{-1} until the fish reached their critical thermal maximum (CT_{max}). These CT_{max} tests were conducted both under normoxic (100% air saturation) and hyperoxic (150% air saturation) conditions. One-hundred and 50% air saturation was chosen as this was the highest oxygen level measured in Page Creek, Cape Eleuthera, The Bahamas (see Figure 1). N = 9. f_H = heart rate; $\dot{Q}$ = cardiac output; S_V = stroke volume; $\dot{M}O_2$ = oxygen consumption; $\dot{M}O_2$ / $\dot{Q}$ = tissue oxygen extraction.

Journal of Experimental Biology • Supplementary information
